# Effect of Novel Processing Techniques on the Carotenoid Release during the Production of Red Guava Juice

**DOI:** 10.3390/molecules29020487

**Published:** 2024-01-18

**Authors:** Xiaoxue Zheng, Ziting Chen, Ziming Guo, Mengting Chen, Bijun Xie, Zhida Sun, Kai Hu

**Affiliations:** 1 College of Food Science and Technology, Huazhong Agricultural University, Wuhan 430070, China; 2 School of Food and Biological Engineering, Jiangsu University, 301 Xuefu Road, Zhenjiang 212013, China

**Keywords:** guava, carotenoid, high-pressure homogenization, enzymatic treatment, pulsed electric field, high hydrostatic pressure

## Abstract

Red guava, distinguished by its elevated lycopene content, emerges as a promising natural source of carotenoids. This study systematically evaluates the impact of diverse processing techniques on the efficient release of carotenoids. The primary objective is to facilitate the transfer of carotenoids into the juice fraction, yielding carotenoid-enriched juice seamlessly integrable into aqueous-based food matrices. The untreated guava puree exhibited a modest release of carotenoids, with only 66.26% of β-carotene and 57.08% of lycopene reaching the juice. Contrastly, both high-pressure homogenization (HPH) at 25 MPa and enzyme (EM) treatment significantly enhanced carotenoid release efficiency (*p* < 0.05), while high hydrostatic pressure (HHP) at 400 MPa and pulsed electric field (PEF) of 4 kV/cm did not (*p* > 0.05). Notably, HPH demonstrated the most substantial release effect, with β-carotene and lycopene reaching 90.78% and 73.85%, respectively. However, the stability of EM-treated samples was relatively poor, evident in a zeta-potential value of −6.51 mV observed in the juice. Correlation analysis highlighted the interactions between pectin and carotenoids likely a key factor influencing the stable dissolution or dispersion of carotenoids in the aqueous phase. The findings underscore HPH as a potent tool for obtaining carotenoid-enriched guava juice, positioning it as a desirable ingredient for clean-label foods.

## 1. Introduction

*Psidium guajava* L., popularly known as guava, belonging to the family of Myrtaceae, is a native species from Mexico and widely cultivated in tropical and subtropical regions [1]. People have been eating guava for more than 2000 years; it can be consumed freshly or processed into various products such as jams, jellies etc. [2]. According to Zheng et al. [3], the main carotenoid in white guava is lutein, while pink/red guava contains mainly lycopene (57.60 µg/g) and β-carotene (14.43 µg/g). The red guava has a short shelf life (3–4 days) for fresh fruit, and due to its high carotenoid content, it can be considered a natural source of carotenoids in the food industry, giving a vibrant red or pink color to foods.

Carotenoids, also known as polyene pigments, are the most studied natural pigments and biosynthesized in almost all fruits and vegetables [4]. The presence of the long chromophore of conjugated double bonds is needed for a carotenoid to have perceptible color [5], and the color of carotenoids changes with the number of conjugated double bonds, usually varying from yellow to red. In addition to bright colors, carotenoids also possess a variety of biological functions, such as pro-vitamin A activity and excellent antioxidant activity [6]. However, the hydrophobic structure of carotenoids makes it difficult to incorporate into aqueous-based food matrices [7]. A carotenoid-rich plant concentrate (juice) that can be stable and evenly dispersed in water-based food matrices can not only serve as an ingredient for clean-label foods but also elevate the health benefits of carotenoids. It is necessary to get rid of the multiple physical barriers in the cell at first to extract carotenoids in red guava. During the production of carotenoid-rich juices, several researchers also described high amounts of carotenoids being retained in the pomace (solid) fraction rather than being transferred into the juice [8,9,10]. Therefore, the production of plant concentrates rich in water-soluble (or well-dispersed) carotenoids with high efficiency, low cost, green, and safe processing procedures is an important issue that needs to be solved.

Emerging technologies, such as high hydrostatic pressure (HHP), high-pressure homogenization (HPH), and pulsed electric field (PEF), can promote the release of small and medium molecules in cells, which have been proven to be quite effective in extracting plant pigments [11]. The high-speed impact force, high-frequency vibration force, cavitation, high shear stress, instantaneous pressure decrease, and high pressure produced by HPH cause changes in food particle structure by disaggregation of cell clusters and disruption of cell structures [12]. According to Chauhan et al. [13], HPH is highly recommended because of its potential to efficiently retain bioactive compounds such as polyphenols, antioxidants, and vitamins in various food products. PEF is a new wall-breaking technique in which samples are exposed to a high-intensity electric field, and voltage is applied in the form of repeated pulses for a few milliseconds to improve the permeability of the cell membrane and promote the release of substances inside the cell [14]. Using water as a medium for pressure transmission, HHP has traditionally been used to inactivate food microorganisms [15]. Recent investigations have shown that it has a substantial force on raw cells, resulting in the rupture of cell walls and facilitating the enhanced release of bioactive compounds [15,16,17]. Enzyme (EM) treatment is often used in juice processing to improve juice yield or to prepare clarified juice. By selecting appropriate enzymes to hydrolyze cell wall components such as pectin and cellulose that hinder the release of intracellular substances [18], the diffusion resistance can be reduced, and the pigment extraction rate can be improved. Atencio et al. [19] also proposed that HPH and EM treatment increased the transfer of carotenoids in pumpkin cells to the juice portion (with carotenoid concentrations in these juices up to 90–98% and 72–90%, respectively) and in pumpkin cells, carotenoids accumulate in chromoplasts in a lipid-soluble form. Nevertheless, carotenoid efficient release assisted with different cell-wall-destruction techniques mentioned above from plant cells in which carotenoids are deposited in a crystalline form (such as guava) and were hardly studied [20].

The juice rich in carotenoids obtained from guava may be a potential alternative for artificial food colorants. The production of high-value-added products with natural colorants has also led to the expansion of guava’s application areas. This study investigated the effect of HPH, EM, PEF, and HHP treatments on the release of carotenoids from guava puree and the physicochemical properties of obtained juices to enhance the release and transfer of carotenoids from guava cells into juice fraction. Exploring high efficiency, low cost, green, and safe methods to obtain water-soluble (dispersed) carotenoid plant concentrates can greatly address the current dilemma of carotenoid application in food. All processing methods were under appropriate parameters determined by the pre-experiments. In industrial production, guava peeling not only makes the process more complicated but also causes production waste. Hence, the effect of guava peel was also studied in this study.

## 2. Results and Discussion

### 2.1. Juice Yield

The effects of different processing techniques on juice yields are shown in Table 1. There was no significant difference in juice yield between GWP and GWOP, indicating that the presence or absence of peel had no effect on the juice yield of guava. Nevertheless, almost all processing techniques increased the juice yields. Specifically, the juice yield in GWOP-E was the highest (85.58%), which was 15.51% higher than that in GWOP. It has been reported that cellulase and pectinase can degrade the cell wall components of fruit by opening the glycosidic linkages, thus increasing the juice yield and enhancing the release of target substances [21,22]. Besides, the degradation of pectin leads to a decreased water-holding capacity of pectin, which releases free water in the system, hence increasing juice yield [22]. HPH treatment also significantly increased the juice yield by 8.21% (*p* < 0.05). It could be ascribed to the disruption of guava cells caused by shearing, cavitation, and turbulence effects from HPH. However, the effects of PEF and HHP treatments on juice yield were limited, though previous studies showed that both two treatments could affect the permeability of cell membrane thus contributing to the extraction of the intracellular substances [10,23,24]. The foregoing results indicated that EM and HPH could be as useful tools to promote the transfer of water-soluble fractions from fruit matrix to juice.

### 2.2. Particle Size Distribution and Zeta Potential

**Figure 1:**
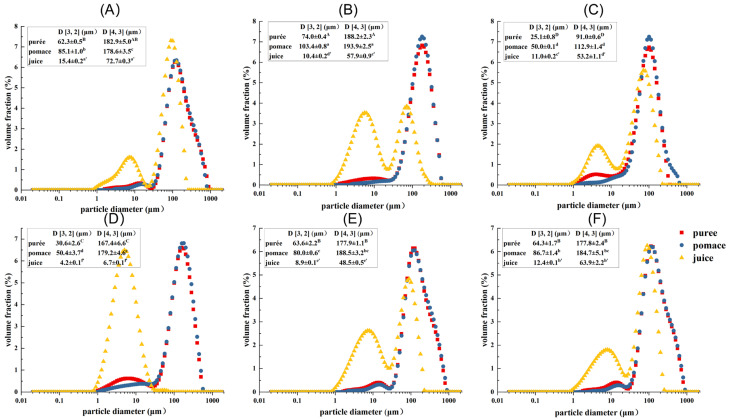
Particle size distribution (PSD) of guava samples. (**A**) Untreated puree with peel (GWP); (**B**) Untreated puree without peel (GWOP); (**C**) GWOP samples treated by HPH; (**D**) GWOP samples treated by EM; (**E**) GWOP samples treated by PEF and (**F**) GWOP samples treated by HHP. ■ puree; ● pomace; ▲ juice. Means with different letters showed significant difference (*p* < 0.05).

The PSD of guava samples treated by different processing techniques are shown in Figure 1. In the disperse system, D [3, 2] and D [4, 3] are mainly related to the particles with smaller and larger particle sizes, respectively [25]. It showed that the juice fractions possessed the lowest D [3, 2] and D [4, 3] values (corresponding to the largest peak area in the range of 1–20 μm), followed by the puree and pomace fractions. This indicated that the juice fractions had more small particles in the range of 1–20 μm, which probably consisted of intracellular substances and small cell fragments. Compared with untreated samples, HPH treatment significantly decreased the particle size of puree and pomace with the D [4, 3] decreased by about 50% (*p* < 0.05), while the particle size of juice fraction was little changed. This indicated that HPH mainly exerted shearing force on large particles, such as cells, cell clusters, and big cell fragments. The D [3, 2] values of puree and pomace were reduced by about 50% upon enzymatic treatment, while the D [4, 3] values changed little. In addition, the particle size of the juice fraction dramatically decreased (88% and 60% for D [4, 3] and D [3, 2], respectively). This indicated enzymatic treatment had a more pronounced effect on small particles. Compared with HPH and enzymatic treatments, the effects of HHP and PEF treatments on particle size were limited, though the particle sizes of puree and pomace were decreased slightly. In addition, the D [3, 2] and D [4, 3] of PEF-Juice also significantly decreased (*p* < 0.05), which might be related to the electroporation under the repeated pulses [24]. However, the D [3, 2] and D [4, 3] of HHP-Juice were higher than GWOP, indicating that the cell wall and cells were broken under high pressure, with some substances released, causing the changes in particle size of the juice [26].

**Figure 2:**
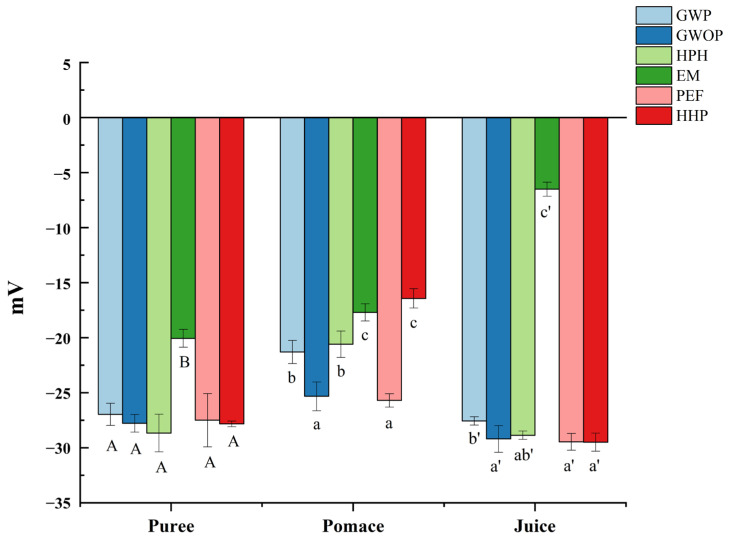
Effect of different treatments on the zeta potential of guava samples. In each component, different letters represent significant differences (*p* < 0.05).

Zeta potential can be used to indicate the degree of electrostatic repulsion between adjacent particles with the same charge in suspension so as to judge the stability of colloidal suspensions [27]. As shown in Figure 2, the particles of all samples (puree, juice and pomace) were negatively charged, which could be ascribed to the pectin layer bound to the surface of particles [28]. Although the zeta potential of purees from GWOP and GWP were similar, the value of GWOP pomace and juice were lower than GWP, which might be related to the pectin in the peel. Compared with the control, HPH, PEF, and HHP treatments had no significant effect on the zeta potential of particles in juice fraction (*p* > 0.05), while significant reduction (about fivefold) was detected in EM-treated samples (*p* < 0.05). It could be speculated that the pectin around particles was degraded under the action of pectinase, leading to the exposure of more positively charged groups [29]. Results indicated that the juice fraction obtained from the control, HPH, PEF, and HHP samples had similar stability, while EM-treated samples showed relatively poor stability. Considering the dispersion and uniform distribution in the food matrix, the stability of natural colorants is often crucial.

### 2.3. Microstructural Characterization

**Figure 3:**
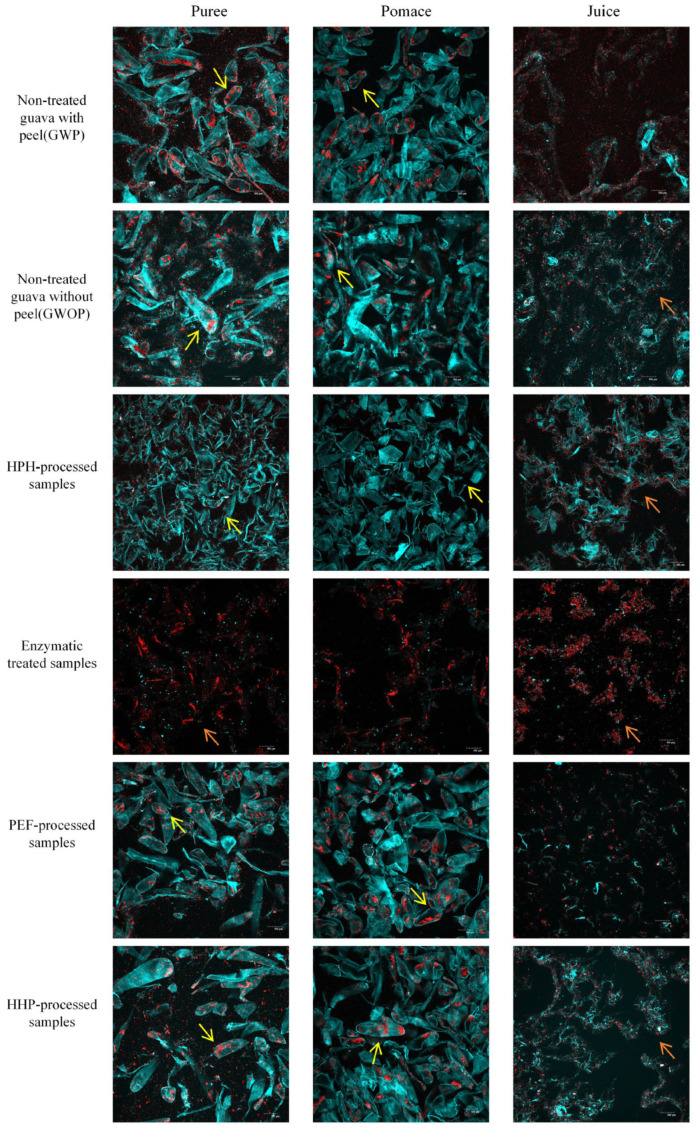
CLSM images of guava samples (puree, pomace, and juice) treated by different processing techniques (yellow arrows point to intact cells, and orange arrows point to discrete carotenoids).

The microstructure of guava samples treated by different processing techniques was observed by CLSM (Figure 3), with the cell wall (blue) and carotenoid (red) being stained. Meanwhile, the mean fluorescence intensity was calculated to quantify the concentration of cell walls and carotenoids (Table 2). There are many intact cells in the puree and pomace of GWP and GWOP, in which a considerable number of big carotenoid aggregates were included, while in the juice fraction, a small amount of dispersed cell wall polymers and carotenoid aggregates with small particle size were observed. Also, no significant difference was detected in the mean fluorescence intensity (blue and red signals) of GWP and GWOP in the juice fraction. This indicated that the presence or absence of guava peel had no significant effect on the transfer of carotenoids and cell wall materials from the fruit matrix to the juice fraction.

In addition, the impact of various processing techniques (HPH, PEF, HHP, and EM treatment) on the release of carotenoids was investigated. Due to the consequent cavitation and high shear stress [30], all intact cells had been destroyed, and cell contents were released after HPH treatment (25 MPa for two passes). After sieving, HPH-pomace contained substantial broken cell wall debris and less dispersed carotenoids. In comparison to GWOP-Juice, HPH-Juice contained more stable cell wall fragments, which formed stronger network structures and were more readily bound to carotenoids, contributing to the stabilization of carotenoids in the aqueous phase. Similarly, Croak et al. [29] described that the negatively charged pectin in orange juice might wrap around small particles, forming a protective layer. Table 2 also showed that the highest concentrations of cell wall polymers (blue signal) and carotenoids (red signal) were detected in HPH-Juice.

The treatment of GWOP with pectinase and cellulase resulted in a complete loss of cell integrity. Pectin is a linear polysaccharide characterized by a high content of GalA, which embeds in cellulose in the cell wall, making cellulose difficult to hydrolysis [31]. Under the synergistic effect of pectinase and cellulase, pectin molecules broke down into smaller oligalacturonans [1], whereas cellulose chains split into glucose, essentially achieving complete liquefaction. There were very few cell wall fragments in the EM-Pomace as well as the EM-Juice, and carotenoids were clumped with the remaining cell wall components in large aggregates. Although the EM resulted in the release of almost all carotenoids from the plant cells, there was no significant increase in the red signal in the EM-Juice (*p* > 0.05). Both blue and red signal values in EM-Juice were significantly lower than in HPH-Juice (*p* < 0.05). This indicated that the solubility or dispersity of carotenoids in the juice phase decreased after pectin was hydrolyzed. It could be concluded that pectin was crucial to the solubility or dispersity of carotenoids in the aqueous phase, and HPH can promote the interaction between pectin and carotenoids, thus increasing the concentration of carotenoids in juice fraction [32].

Individual cells were still very visible in PEF-treated and HHP-treated purees and pomace, with the crystalline chromoplasts containing carotenoids still inside the cells, similar to the GWP and GWOP samples. After sieving, only a few scattered cell wall fragments and released carotenoids were visible in PEF-Juice. Under sufficient electric fields, a phenomenon called electroporation occurs, leading to the increment of the permeability of the cytoplasmic membrane to the passage of ions and macromolecules [33]. As previous study, the matrix and cell disruption were rather limited in PEF compared with HPH-treated samples [10,19,24]. Although HHP treatment caused changes in membrane permeability, destruction of cell walls, and higher extraction rates for metabolite analysis [23,34], a limited effect on cell integrity was observed, and many carotenoids were trapped within cells. This indicated that static high pressure could not effectively improve the transfer of carotenoids into the aqueous phase.

### 2.4. Effect of Processing Treatments on Carotenoid Contents of Puree, Pomace, and Juice

A previous study has isolated and identified 16 carotenoids from the flesh of Brazilian red guavas [35]. Zheng et al. [3] confirmed (E/Z)-phytoene, lycopene, and β-carotene (>85%) as major carotenoids in red and pink guava. Rojas-Garbanzo et al. [36] also reported that the all-trans-lycopene was found to be predominant (from 63% to 92% of total carotenoids) in pink guava. In this study, only β-carotene and lycopene were quantitatively analyzed since they accounted for over 82% of the total carotenoid content (as shown in Supplementary Materials).

**Figure 4:**
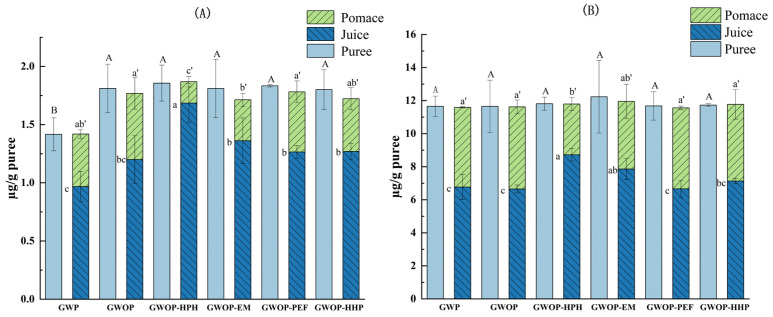
Carotenoid contents of different treated guava samples (the carotenoid contents in juice and pomace were presented per gram of the corresponding purees). (**A**) β-carotene; (**B**) lycopene. In each figure, different letters represent significant differences (*p* < 0.05).

Figure 4 presents the effect of different treatments on the carotenoid contents of guava puree as well as the corresponding resulting juice and pomace. Firstly, the effect of the raw material (presence or absence of guava peel) on the transfer of carotenoids from puree to pomace and juice fractions was explored. The amount of β-carotene was significantly higher in GWOP compared to GWP (Figure 4A) (*p* < 0.05), while the lycopene content showed no significant difference between GWOP and GWP (Figure 4B). This might be related to the lower carotenoid content in the peel. Upon sieving, the amount of total carotenoid transferred into GWP-Juice was 7.74 ± 0.89 µg/g, while about 41% was retained in GWP-Pomace. Likewise, the total carotenoid content of GWOP-Juice was 7.85 ± 0.44 µg/g. This indicated that the presence or absence of peel could hardly affect the transfer of carotenoids from the solid phase (pomace) to the aqueous phase (juice).

As shown in Figure 4, there were no significant differences in carotenoid contents (β-carotene and lycopene) between guava purees treated by different processing techniques (*p* > 0.05), indicating that the effect of these processing techniques on carotenoid content was negligible. Compared with the control, HPH treatment at 25 MPa for two passes increased β-carotene and lycopene contents of the HPH-Juice by 40.8% and 31.1%, respectively, indicating that HPH played a favorable role in promoting the transfer of carotenoids from solid phase to aqueous phase. Under the action of HPH, due to the sudden formation of turbulence, shear stress, and cavitation [17], the cell wall and cell membrane are subjected to strong mechanical damage, thus promoting the release of carotenoids. Due to the fat-soluble structure, carotenoids are hydrophobic; thus, their dispersibility in the juice phase indicated an alteration of the chromoplast and formation of micellar structures [8,37]. The result implied that carotenoids were likely to be complexed or associated with hydrocolloids present (e.g., proteins, pectin) as colloidal particles [8,37]. The combination of lycopene and soluble pectin has been confirmed during tomato processing by Jazaeri et al. [38], which increased the solubility of lycopene. Recently, Hu et al. [32] also speculated that HPH increased the solubilization (or dispersion) of carotenoids in mango juice through carotenoid-pectin interaction. Therefore, available data suggested that HPH has the potential to improve the transfer of carotenoids to the aqueous phase by causing cell wall fragments to form a more stable network structure, thereby facilitating the binding of carotenoids to hydrophilic colloids (pectin).

In the food industry, cellulase and pectinase were usually used to liquefy fruit and vegetable purees to break down cell walls and extract pigments [22]. As exhibited in Figure 4, after the EM treatment, the carotenoid content in the juice also increased compared to the control, and about 75% β-carotene and 64% lycopene were transferred into the juice phase. A similar effect was noted previously by Atencio et al. [19] for experiments done with pumpkin, resulting in a significant increase in carotenoid contents in the juice (72–90%). However, the β-carotene and lycopene contents in EM-Juice were lower than in HPH-Juice. The hydrolysis of pectin might not be conducive to the dissolution and dispersion of carotenoids in aqueous solutions, resulting in a certain degree of carotenoid aggregation, which was allocated to pomace during filtration.

The contents of two carotenoids in PEF-Juice and HHP-Juice were lower than those in HPH-Juice, just slightly higher (not significantly) than that in GWOP-Juice. Only 69.4% of β-carotene and 60.8% of lycopene in GWOP-PEF were transferred to PEF-Juice. The quantitative results were consistent with the observations by CLSM, indicating that PEF treatment at the chosen parameters had a limited effect on the release of carotenoids from cells. In previous studies, others had reported similar effects of PEF in experiments with carrot juice [9] and pumpkin puree [19]. On the one hand, it might be due to the low electric field intensity (4 kV/cm) used in the experiment. Several studies have shown that at higher electric field strengths (35–40 kV/cm) and longer treatment times, PEF enhanced the yield of carotenoids in orange-carrot juice and other mixed juices [24,39]. In addition, the substructure of the chromoplast containing carotenoids in guava cells presents in a crystalline form, which might be unfavorable to its release across the cellular barrier. The contents of β-carotene and lycopene in HHP-Juice were 1.27 ± 0.07 µg/g puree and 7.13 ± 0.16 µg/g puree, which showed no significant difference compared with the control. As a widely used non-thermal processing technology in the past, HHP has been used primarily to inactivate microorganisms in food systems [40]. In recent years, the role of HHP as a pretreatment technique to facilitate the retention and extraction of potentially health-related compounds has received extensive attention from researchers [41]. The most widely supported theory is that HHP treatments (≥200 MPa) alter membrane permeability and disrupt cell walls, thereby improving the analytical extraction of metabolites [34,42,43]. Many studies have also shown that HHP can be used as a suitable treatment for increasing the extraction of carotenes from the matrix [23,44]. It has also been reported that HHP improved the bioaccessibility of carotenoids in persimmon pulp, which may be due to the structural changes of pectin produced by HPP and the interaction with other plant tissue components [26]. However, according to the CLSM image, HHP of 400 MPa for 20 min did not effectively destroy the cell wall. As mentioned before, Hu et al. [32] conducted HPH and HHP on mango juice and found that HPH had a more obvious effect on WSP than HHP due to stronger mechanical force, while HHP alone had a slight increase in GalA concentration and WSP molecular weight (Mw) in mango juice. Therefore, the efficacy of HHP in cell wall disruption was limited, resulting in the incomplete release of carotenoids.

**Figure 5:**
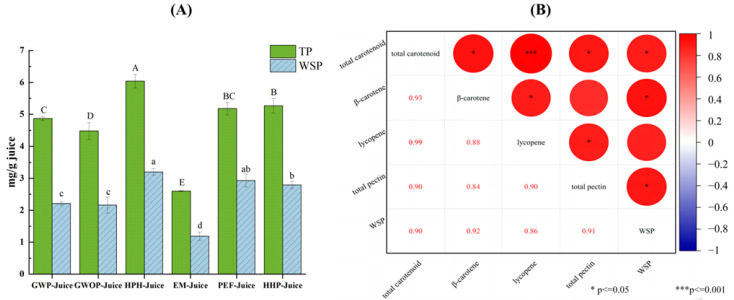
(**A**) Total pectin and water-soluble pectin content of guava juices. (**B**) Pearson correlation analysis of pectin contents and carotenoid contents in juice fraction. In Figure (**A**), different letters represent significant differences (*p* < 0.05).

Figure 5 presents the effects of different treatments on pectin contents in juice fraction and the relationship between pectin and the enrichment of carotenoids in juice fraction. Due to the high pectin content in the peel, the total pectin content of GWP-Juice was found to be higher than that of GWOP, but there was no significant difference in WSP content (*p* > 0.05). Compared with other groups, the contents of total pectin and WSP in HPH-Juice were the highest, indicating HPH could significantly increase the release of pectin from the solid fraction (cell wall) to the juice fraction (*p* < 0.05). Similar findings were also found in mango juice treated by high pressure [32]. The increases both in total pectin and WSP were also detected in PEF-Juice and HHP-Juice. Correlation analysis showed that total carotenoid content was significantly correlated (r = 0.90, *p* < 0.05) with total pectin and WSP, respectively. Significant correlations were also found between β-carotene and WSP (r = 0.92, *p* < 0.05), as well as lycopene and total pectin (r = 0.90, *p* < 0.05), respectively. This indicated that interaction between pectin and carotenoids induced by different processing techniques may be crucial to the enrichment of carotenoids in juice fraction. The contents of total pectin and WSP in EM-Juice were the lowest due to the action of pectinase. Although the carotenoid content in EM-Juice was higher than control, the formation of large aggregates (fluorescence microscope observation) and the reduction of stability (Zeta-potential analysis) were found in Section 3.2 and Section 3.3. This indicated pectin played significant roles not only in carotenoid enrichment but also in the dispersion stability of carotenoids in juice fraction.

### 2.5. Compositional Analyses and PCA of Samples

Table 3 shows the determination of some basic components and properties of guava samples. Crude lipid, total protein, and ash contents were mostly similar in GWP, GWOP, and processed puree. However, the contents of these components vary apparently in juices and pomace. As anticipated, the crude lipid and protein contents of HPH-Juice had the most predominate increase, and its crude lipid content reached 6.01 ± 0.17 g/100 g puree, which was about 4.5 times higher than that of GWOP, possibly relating to its high carotenoid content. As speculated in many studies [37,38], it can be considered that fat-soluble are likely to be associated with hydrocolloids (e.g., proteins, pectins) present as colloidal particles, thereby enhancing their distribution in the aqueous phase. In line with the study of Juric et al. [8], after only HPH for one pass, the total released proteins increased by 41.9% with respect to control, whereas after 10 passes, they increased by 70.5%. Similar results were obtained from the pumpkin puree [19].

Similarly, pH, TSS, and color of guava samples were also determined. EM treatment of GWOP greatly decreased the pH value of EM-Juice. Through the study of enzyme concentration, the hydrolysis of pectin by pectinase is accompanied by the liberation of GalA and of carboxylic acid from GalA upon demethylation [45]. The pH values of the other treated/non-treated groups (including GWP) were similar, ranging from 5.33 to 5.29, indicating that these treatments did not release acidic compounds into the juice.

TSS was determined for juice samples only. HPH-Juice had the highest TSS of 1.32 ± 0.03 ^◦^Brix, while EM-Juice had a slightly lower TSS than HPH-Juice at 1.21 ± 0.05 ^◦^Brix. The higher TSS might be related to the fact that both treatments caused the greatest disruption of the cells, facilitating the release of soluble solids. Moreover, the release of some neutral sugars after degradation of cell wall pectin, cellulose and hemicellulose, or the assimilation of the released galacturonic monomers into glucose molecules could also be explained by the effect of EM treatment [1].

The color of guava samples is reported as CIE L* (lightness), a* (redness: green to red), and b* (yellowness: blue to yellow) values. The use of the CIELAB color system has been proven to provide thorough coverage of perceptual attributes necessary to examine the color of food products [46]. For natural colorants, good color is a necessary condition for a wide range of applications, which is usually related to the amount of pigment present in the material [47]. According to Gliemmo et al. [47], the values of a* and b* are related to carotenoid concentrations. After processing, the L* and a* values of HPH-Juice and EM-Juice were significantly higher than those of other treatment groups (*p* < 0.05), indicating that the juices were brighter and redder, and more carotenoids (especially lycopene) were transferred to the HPH-Juice and EM-Juice. In contrast, the changes in PEF and HHP samples were not obvious, which was consistent with the results of carotenoid content determination. The effect of HPH on juice color, especially on the increase in the red component (a*), can be explained by disruption of the cells and membranes and breakage of the chromoplast carotenoid–protein complexes during processing, allowing for the leakage of pigments such as lycopene [48]. Moreover, the increase in conductivity and color of guava juice caused by EM treatment has also been reported [1,49]. Overall, the higher carotenoid content of HPH-Juice and EM-Juice have a higher relative brightness and redder color, which would be in line with the market’s expectations for natural carotenoid pigments.

**Figure 6:**
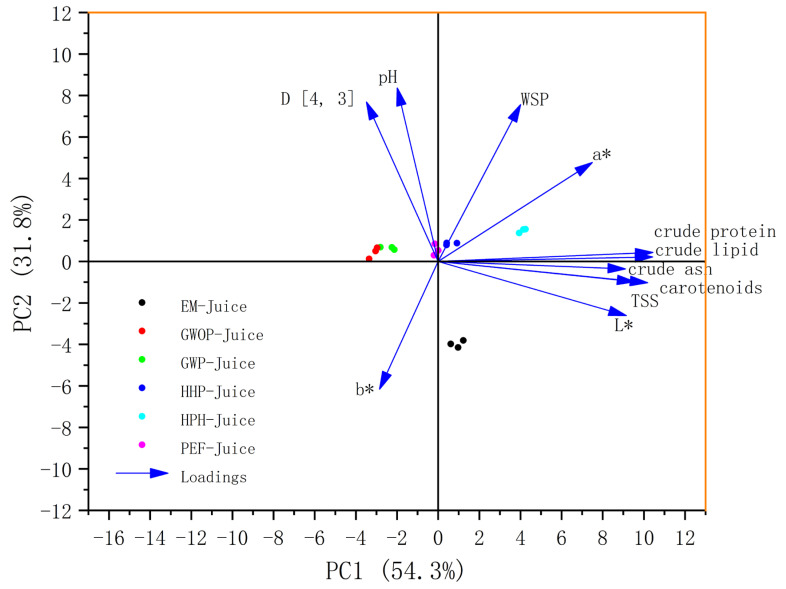
PCA score diagram of different treated guava juices.

PCA is an unsupervised chemometric technique that can be used to reveal the hidden trends in sample clustering. As shown in Figure 6, the first two principal components (PCs) explained 87.6% of the total variance, indicating that these two PCs included most information of quality variation. In this plot, all the parameters, namely carotenoid content, TSS, pH, D [4, 3], WSP, basic components, and CIE values score, were denoted as loadings. Most of the parameters showed positive correlations with PC1, with the basic components, carotenoid content, TSS and L* having a large contribution to PC1. Among all the parameters, only b* showed a negative correlation with both PC1 and PC2. The samples lying close to each other in the bi-plot are often characterized by similar properties. For example, the GWOP-Juice and GWP-Juice points were close, suggesting that the presence of peel did not cause significant changes in juice characteristics. Moreover, all treated sample points shifted in the positive direction of PC1, with HPH-Juice moving the most. On the contrary, in terms of PC2, the EM-Juice showed a significant negative shift, implying that the EM treatment drastically altered the relevant properties of the juice.

## 3. Materials and Methods

### 3.1. Materials

Fully ripe red guava fruits (*Psidium guajava*) used for the experiments were purchased from an orchard in Zhangzhou, China, in November 2022. Guavas were selected based on their degree of maturity (eating stage) and processed within 3 days upon arrival. All chemicals used were of analytical grade. Lycopene standard (purity ≥ 95), β-carotene standard (purity ≥ 98) and other chemicals used for carotenoid quantification were of HPLC grade. Pectinase (isolated from *Aspergillus niger*, activity: 30,000 U/g) was obtained from Shanghai Macklin Biochemical Technology Co., Ltd. (Shanghai, China). Cellulase (isolated from *Trichoderma viride*, activity: 40,000 U/g) was obtained from Shanghai Yuanye Bio-Technology Co., Ltd. (Shanghai, China).

### 3.2. Preparation of Guava Purees, Pomace, and Juices

#### 3.2.1. Preparation of Guava Purees

A puree with peel (GWP) and a puree without peel (GWOP) were prepared by slicing guava (with peel or without peel) and blending with demineralized water (1:3 *w*/*w* guava:water, based on wet weight). The mixture was blended 3 times for 10 s (Mixer AUX-PB593, Haixun Electric Co., Ltd., Foshan, China), and the guava seeds were removed through a 100-mesh sieve to prevent any impact on subsequent processing and testing, resulting in GWP and GWOP. Within 10 min after sieving, the purees were further separated into a juice and pomace fraction by a 400-mesh sieve. The collected juice fractions were not subjected to any concentration process. The puree and collected fractions were frozen in liquid nitrogen and stored at −80 °C until analysis.

#### 3.2.2. High-Pressure Homogenization

The HPH-processed puree was prepared by GWOP after HPH (AH-Basic, ATS Engineering Co., Ltd., Suzhou, China) at 25 MPa for 2 passes. Within 10 min after processing, the puree was separated into a juice (HPH-Juice) and pomace (HPH-Pomace) fraction by a 400-mesh sieve. The puree and collected fractions were frozen in liquid nitrogen and stored at −80 °C until analysis.

#### 3.2.3. Enzymatic Treatment

The pH of GWOP was adjusted to 4.6 (suitable pH for pectinase and cellulase) with 0.1 mol/L HCl, and then 0.02% pectinase and 0.01% cellulase were added. Next, the mixture was incubated in a water bath with a shaking plate at 37 °C for 1 h. Within 10 min after processing, the purees were separated into a juice (EM-Juice) and pomace (EM-Pomace) fraction by a 400-mesh sieve. The puree and collected fractions were frozen in liquid nitrogen and stored at −80 °C until analysis.

#### 3.2.4. Pulsed Electric Field Treatment

Pulsed electric field treatment was carried out by THU-PEF4 device (Wuhan Polytechnic University, Wuhan, China). The electrode diameter was 0.3 cm, and the gap distance was 0.3 cm. GWOP-PEF was prepared with a fixed electric field intensity of 4 kV/cm, a frequency of 1.06 kHz, a pulse width of 40 µs, a flow rate of 0.8 mL/s and 5 cycles. Within 10 min after processing, the purees were separated into a juice (PEF-Juice) and pomace (PEF-Pomace) fraction by a 400-mesh sieve. The puree and collected fractions were frozen in liquid nitrogen and stored at −80 °C until analysis.

#### 3.2.5. High Hydrostatic Pressure

The GWOP puree was packaged in food PE bags and treated by HHP (BaoTou KeFa High Pressure Technology Co., Ltd., Baotou, China) at 400 MPa for 20 min, and the pressure relief time was about 10~20 s to obtain the GWOP-HHP. Within 10 min after processing, the purees were separated into a juice (EM-Juice) and pomace (EM-Pomace) fraction by a 400-mesh sieve. The puree and collected fractions were frozen in liquid nitrogen and stored at −80 °C until analysis.

### 3.3. Mass Yield and Microstructural Analyses

#### 3.3.1. Mass Yield of Juice and Pomace Samples

The juice and pomace fractions obtained were calculated on a mass basis as described below in Equations (1) and (2), respectively.

(1) $$\mathrm{Juice}\;\mathrm{yield}(\%)=\frac{\mathrm{mass}\;\mathrm{of}\;\mathrm{juice}\;(g)}{\mathrm{mass}\;\mathrm{of}\;\mathrm{corresponding}\;\mathrm{puree}\;(g)}\times 1$$

(2) $$\mathrm{Pomace}\;\mathrm{yield}(\%)=\frac{\mathrm{mass}\;\mathrm{of}\;\mathrm{pomace}\;(g)}{\mathrm{mass}\;\mathrm{of}\;\mathrm{corresponding}\;\mathrm{puree}\;(g)}\times 1$$

#### 3.3.2. Confocal Laser Scanning Microscopy (CLSM)

According to the methods reported by Jianing et al. [50], the microscopic morphology of guava samples was observed using CLSM (FV3000, Olympus Corporation, Tokyo, Japan). The carotenoids and cell walls were stained by adding Nile red (1 mg/mL) and Calcofluor white (1 mg/mL). All pomace samples were diluted with demineralized water (1:1 *v*/*v*) before dyeing. The dispersion of carotenoids and cell wall breakage were observed at the corresponding excitation wavelength.

#### 3.3.3. Particle Size Distribution (PSD)

The PSD was determined using the Mastersizer 2000 laser particle size distributor (Malvern Instruments, Malvern, UK), with a detection range of 0.02 to 2000 µm. The volume-mean diameter (D [4, 3]) and area-mean diameter (D [3, 2]) of all samples were calculated by the software provided by the device. All tests were performed in triplicate.

#### 3.3.4. Zeta Potential

The sample was diluted with ultrapure water, and the zeta potential was determined by Nano-ZS-90 (Malvern Instruments, Malvern, UK) at 25 °C. All tests were performed in triplicate. Data were collected and analyzed using Zetasizer software v7.02.

### 3.4. Carotenoid Extraction and HPLC Analysis

#### 3.4.1. Carotenoid Extraction

Carotenoids were extracted according to the procedure of Wibowo et al. [51]: the sample was mixed with 15 mL extraction solvent (50:25:25 hexane:ethanol:acetone (*v*/*v*/*v*) containing 0.1% butylated hydroxytoluene (*w*/*v*)). After mixing thoroughly, 5 mL reagent-grade water and 0.5 g NaCl were added, and the mixture was stirred for 20 min. The mixture was allowed to separate into two layers in a covered glass tube. The upper carotenoid-containing layer was collected, while the lower layer was re-extracted with the same extraction solvent until the lower layer became clear.

The carotenoid-containing hexane layer was collected and concentrated in a 35 °C rotary evaporator for 10 min. The residue was redissolved in 1 mL dichloromethane (containing 0.1% butylated hydroxytoluene (*w*/*v*)), and the sample was filtered in a 0.22 µm pore size and added to vials. All samples were extracted in triplicate and under subdued light to avoid carotenoid degradation.

#### 3.4.2. Determination of Carotenoids by High Performance Liquid Chromatography (HPLC)

Carotenoids were analyzed with the e2695 HPLC system (Waters Corp., Milford, MA, USA), equipped with a C30-column (250 mm × 4.6 mm, 5 µm, YMC Europe, Dinslaken, Germany), coupled to a corresponding C30 guard cartridge. The column was maintained at 25 °C, with methanol (A) and MTBE (B) used as mobile phase. Gradient elution was applied at a flow rate of 1 mL/min with the following conditions: 0–15 min 80% A + 5% B, 20 min 65% A + 35% B, 24 min 35% A + 65% B, 30 min 32% A + 68% B, 37 min 20% A + 80% B, 39 min 10% A + 90% B, 43–45 min 80% A + 20% B. The volume of injection ranged was 40 µL. The wavelengths were set to 450 nm and 470 nm.

### 3.5. Compositional Analyses and Quality Attributes of Samples

#### 3.5.1. Determination of the Dry Matter, Total Lipid, Total Protein, and Ash Content

Dry matter content was determined using a vacuum oven (GZX-9140 MBE, Boxun Medical Biological Instrument Co., Ltd., Shanghai, China). Soxhlet extraction method was used to measure the crude lipid. Determination of total protein using the Kjeldahl method is commonly and officially recognized as a standard method [52]. Total protein content was determined using an automatic Kjeldahl nitrogen analyzer (NKB3000, Shanghai Wanghai Environmental Technology Co., Ltd., Shanghai, China) after multiplying the total nitrogen content with an overall conversion factor of 6.25. Total ash content was determined in a muffle furnace (SX2-4-10A, Jiecheng experimental Instrument Co., Ltd., Shanghai, China) for 8 h at 550 °C. The compositional analysis was done in triplicate from a single batch of prepared samples.

#### 3.5.2. Determination of pH and Total Soluble Sugars (TSS)

The pH was measured using a tester (FE30–Five Easy, Mettler Toledo Technology (China) Co., Ltd., Shanghai, China) at room temperature (20 ± 2 °C). TSS in all samples was determined with a handheld refractometer (WYT-15, Taian Hongyi Mining Technology Co., Ltd., Taoyuan, China).

#### 3.5.3. Determination of Color

A Spectrocolorimeter (UltraScan VIS, HunterLab, Reston, VA, USA) was used to measure the color of samples, where L* corresponds to lightness, a* to redness (from green to red) and b* to yellowness (from blue to yellow).

#### 3.5.4. Determination of Total Pectin and Water-Soluble Pectin Content (WSP)

The content of total pectin and water-soluble pectin are expressed as the content of galacturonic acid (GalA). The pectin was extracted from juice in accordance with the method proposed by McCready et al. [53]. Pectin was homogenized in 80% ethanol using 10.00 g juice, and the sample was washed twice with 80% ethanol after being left under light protection for about 12 h overnight. The pH of total pectin was adjusted to 11.5 with 1.0 mol/L NaOH solution for 30 min and then adjusted to 5.0–5.5 with glacial acetic acid to achieve ideal hydrolysis conditions. The alcohol-insoluble residue (AIR) was isolated; first, it was kept in hot water (100 mL) for 5 min, cooled immediately, and filtered by using a Buchner funnel under vacuum and the volume was adjusted to 100 mL with distilled water. The samples were completely hydrolyzed by sulphuric acid-sodium tetraborate solution (0.0125 mol/L sodium tetraborate in 98% H_2_SO_4_) followed by colorimetric analysis with m-hydroxydiphenyl solution (0.15% 3-phenylphenol in 0.50% NaOH) using a spectrophotometer (UV-1800, Shimadzu, Japan) at a wavelength of 520 nm.

### 3.6. Statistical Analysis

Results were presented as a mean ± standard deviation of three replicates performed on each batch of (non)processed samples. The experimental subsequent data processing, a correlation heatmap and principal component analysis (PCA) were executed using the Origin 2021 software. The software IBM SPSS Statistics version 23 (IBM SPSS, Chicago, IL, USA) was used to analyze the data. The analysis of variance (ANOVA) followed by a multiple comparison Tukey HSD (*p* < 0.05) test was conducted.

## 4. Conclusions

This study investigated the effect of the raw material, HPH, cell-wall-degrading EM, HHP, and PEF treatments on the efficient release of carotenoids from guava puree to produce carotenoid-enriched juice, which might be a potential alternative in food coloring. HPH and EM treatments notably increased juice yield, as well as the release of carotenoids and other intracellular components (lipid, protein, and mineral salts) and TSS. However, juice fraction from EM-treated samples presented large carotenoid aggregates and low dispersion stability. HHP and PEF treatments had no significant effect on the transfer of carotenoids into the juice (*p* > 0.05) but promoted the release of other intracellular components (not as many as HPH and EM). Correlation analysis highlighted that pectin released into the juice was a potential key factor for carotenoids’ stability, and the interaction mechanism between pectin and carotenoids needs to be further studied. In conclusion, HPH-assisted processing treatment is a promising approach to producing carotenoid-enriched juice from guava, facilitating the increased use of carotenoids in water-based food products and simultaneously meeting consumers’ demands for clean-label food. In addition, more industrial comparative studies to validate these results and further optimization of the treatment process and conditions are needed in the future.

## Figures and Tables

**Table 1:** Juice yield of guava upon different processing techniques.

Treatments	Puree Weight/g	Pomace Weight/g	Juice Weight/g	Juice Yield (%)
GWP	200.00	50.07 ± 1.42	150.86 ± 1.42	75.08 ± 0.71 ^cd^
GWOP	200.00	51.59 ± 2.96	148.54 ± 2.96	74.09 ± 1.48 ^d^
GWOP-HPH	200.00	39.38 ± 0.91	160.62 ± 0.91	80.17 ± 0.46 ^b^
GWOP-EM	200.00	28.19 ± 0.37	171.81 ± 0.37	85.58 ± 0.19 ^a^
GWOP-PEF	200.00	49.57 ± 1.68	150.64 ± 1.68	75.24 ± 0.84 ^cd^
GWOP-HHP	200.00	48.40 ± 1.04	151.76 ± 1.04	75.82 ± 0.52 ^c^

Means with different letters showed significant difference (*p* < 0.05).

**Table 2:** Mean fluorescence intensity of different treatment groups.

	Blue Signal/AU	Red Signal/AU
Puree		
GWP	73.00 ± 3.57 ^A^	29.49 ± 3.57 ^a^
GWOP	64.30 ± 4.17 ^B^	31.95 ± 3.42 ^a^
GWOP-HPH	61.39 ± 3.76 ^B^	28.50 ± 1.26 ^a^
GWOP-EM	25.18 ± 0.68 ^C^	21.63 ± 2.41 ^b^
GWOP-PEF	64.99 ± 3.12 ^B^	32.32 ± 2.82 ^a^
GWOP-HHP	59.12 ± 4.10 ^B^	28.97 ± 2.03 ^a^
Pomace		
GWP	68.88 ± 1.63 ^A^	23.93 ± 5.39 ^ab^
GWOP	60.05 ± 5.39 ^B^	23.58 ± 1.04 ^ab^
GWOP-HPH	68.28 ± 0.52 ^A^	20.03 ± 1.92 ^c^
GWOP-EM	30.39 ± 0.88 ^C^	21.28 ± 1.56 ^bc^
GWOP-PEF	74.98 ± 3.03 ^A^	24.96 ± 1.90 ^a^
GWOP-HHP	72.97 ± 3.18 ^A^	24.44 ± 1.03 ^a^
Juice		
GWP	26.93 ± 0.08 ^C^	26.57 ± 0.80 ^b^
GWOP	27.96 ± 3.15 ^C^	25.62 ± 0.37 ^b^
GWOP-HPH	44.97 ± 3.86 ^A^	31.10 ± 2.58 ^a^
GWOP-EM	17.42 ± 0.82 ^D^	25.26 ± 3.44 ^b^
GWOP-PEF	18.96 ± 1.46 ^D^	24.33 ± 3.84 ^b^
GWOP-HHP	33.08 ± 2.33 ^B^	27.28 ± 1.14 ^ab^

Means with different letters for each row showed significant difference (*p* < 0.05).

**Table 3:** Proximate composition and quality parameters of guava samples. Values for proximate composition are expressed as g/100 g dry weight of the corresponding puree. n.a. means not analyzed.

	Crude Lipid	Crude Protein	Crude Ash	pH (22 °C)	TSS (22 °C)	CIE Color Values		
						L*	a*	b*
Purees								
GWOP	8.39 ± 0.78 ^a^	13.68 ± 1.26 ^b^	7.90 ± 0.13 ^a^	5.26 ± 0.01 ^ab^	n.a.	38.62 ± 0.41 ^bc^	5.42 ± 0.12 ^c^	0.63 ± 0.04 ^c^
GWP	7.79 ± 0.60 ^a^	12.97 ± 0.41 ^b^	7.42 ± 0.12 ^b^	5.25 ± 0.01 ^ab^	n.a.	38.43 ± 0.19 ^c^	5.11 ± 0.05 ^d^	1.19 ± 0.11 ^ab^
GWOP-HPH	8.63 ± 0.32 ^a^	14.84 ± 0.67 ^a^	7.82 ± 0.06 ^a^	5.28 ± 0.02 ^a^	n.a.	39.07 ± 0.05 ^ab^	5.94 ± 0.15 ^b^	0.76 ± 0.11 ^c^
GWOP-EM	8.48 ± 0.53 ^a^	13.15 ± 0.05 ^b^	7.87 ± 0.35 ^a^	4.17 ± 0.02 ^c^	n.a.	39.23 ± 0.21 ^a^	6.18 ± 0.07 ^a^	1.02 ± 0.11 ^b^
GWOP-PEF	8.50 ± 0.45 ^a^	13.32 ± 0.14 ^b^	7.90 ± 0.27 ^a^	5.24 ± 0.01 ^b^	n.a.	38.42 ± 0.16 ^c^	5.84 ± 0.02 ^b^	1.24 ± 0.11 ^a^
GWOP-HHP	8.46 ± 0.21 ^a^	13.69 ± 0.09 ^b^	7.92 ± 0.04 ^a^	5.26 ± 0.02 ^ab^	n.a.	38.14 ± 0.44 ^c^	5.94 ± 0.14 ^b^	1.25 ± 0.13 ^a^
Juices								
GWOP-Juice	1.40 ± 0.10 ^d^	4.26 ± 0.75 ^d^	3.28 ± 0.02 ^d^	5.31 ± 0.01 ^a^	0.94 ± 0.02 ^c^	37.90 ± 0.16 ^bc^	5.41 ± 0.07 ^c^	1.53 ± 0.07 ^b^
GWP-Juice	1.10 ± 0.06 ^d^	4.03 ± 0.02 ^d^	3.29 ± 0.07 ^d^	5.33 ± 0.01 ^a^	0.87 ± 0.01 ^d^	37.61 ± 0.19 ^c^	4.48 ± 0.16 ^e^	0.73 ± 0.19 ^c^
HPH-Juice	6.01 ± 0.17 ^a^	9.61 ± 0.38 ^a^	5.65 ± 0.03 ^a^	5.31 ± 0.01 ^a^	1.32 ± 0.03 ^a^	38.79 ± 0.03 ^a^	6.15 ± 0.08 ^a^	0.25 ± 0.07 ^d^
EM-Juice	3.54 ± 0.22 ^b^	6.77 ± 0.13 ^b^	5.08 ± 0.03 ^c^	4.22 ± 0.02 ^b^	1.21 ± 0.05 ^b^	38.60 ± 0.15 ^a^	4.81 ± 0.14 ^d^	2.12 ± 0.13 ^a^
PEF-Juice	2.85 ± 0.47 ^c^	5.81 ± 0.07 ^c^	5.36 ± 0.02 ^bc^	5.29 ± 0.01 ^a^	1.16 ± 0.01 ^b^	37.87 ± 0.16 ^bc^	5.45 ± 0.10 ^bc^	1.43 ± 0.12 ^b^
HHP-Juice	3.03 ± 0.19 ^c^	6.98 ± 0.03 ^b^	5.60 ± 0.02 ^b^	5.31 ± 0.01 ^a^	1.18 ± 0.02 ^b^	38.05 ± 0.30 ^b^	5.65 ± 0.12 ^b^	1.42 ± 0.23 ^b^
Pomace								
GWOP-Pomace	6.82 ± 0.16 ^a^	8.87 ± 0.43 ^a^	4.48 ± 0.06 ^a^	5.21 ± 0.01 ^a^	n.a.	41.26 ± 0.15 ^bc^	10.11 ± 0.17 ^b^	2.99 ± 0.13 ^c^
GWP-Pomace	6.57 ± 0.78 ^a^	9.15 ± 0.07 ^a^	4.50 ± 0.02 ^a^	5.22 ± 0.02 ^a^	n.a.	41.73 ± 0.12 ^bc^	7.56 ± 0.23 ^d^	1.94 ± 0.02 ^d^
HPH-Pomace	1.56 ± 0.41 ^c^	4.67 ± 0.14 ^d^	1.96 ± 0.37 ^c^	5.21 ± 0.01 ^a^	n.a.	44.16 ± 0.10 ^a^	3.79 ± 0.04 ^e^	2.63 ± 0.24 ^c^
EM-Pomace	4.93 ± 0.09 ^b^	6.50 ± 0.29 ^bc^	2.36 ± 0.03 ^b^	4.15 ± 0.02 ^b^	n.a.	44.13 ± 0.21 ^a^	14.74 ± 0.24 ^a^	5.00 ± 0.26 ^a^
PEF-Pomace	5.24 ± 0.83 ^b^	6.79 ± 0.15 ^b^	2.14 ± 0.01 ^bc^	5.19 ± 0.01 ^a^	n.a.	42.16 ± 0.27 ^b^	9.95 ± 0.31 ^bc^	3.66 ± 0.08 ^b^
HHP-Pomace	5.08 ± 0.52 ^b^	6.26 ± 0.12 ^c^	2.25 ± 0.02 ^b^	5.22 ± 0.02 ^a^	n.a.	40.95 ± 1.26 ^c^	9.39 ± 0.76 ^c^	3.51 ± 0.60 ^b^

In each fraction, means with different letters for each column showed significant difference (*p* < 0.05).

## Data Availability

Data are contained within the article and Supplementary Materials.

## Supplementary Materials

The following supporting information can be downloaded at: https://www.mdpi.com/article/10.3390/molecules29020487/s1, Figure S1: HPLC-DAD chromatogram of carotenoids detected in the guava samples at 450 nm; Figure S2: HPLC-DAD chromatogram of carotenoids detected in the guava samples at 470 nm.
