# Supplementary material for: Effect of Novel Processing Techniques on the Carotenoid Release during the Production of Red Guava Juice
Source: Molecules. 2024 Jan 18;29(2):487. doi: 10.3390/molecules29020487 (PMC10818855; doi:10.3390/molecules29020487)
Supplement: Supplementary file 1 [file molecules-29-00487-s001.zip › molecules-2824380-supplementary.pdf]

Supplementary Materials

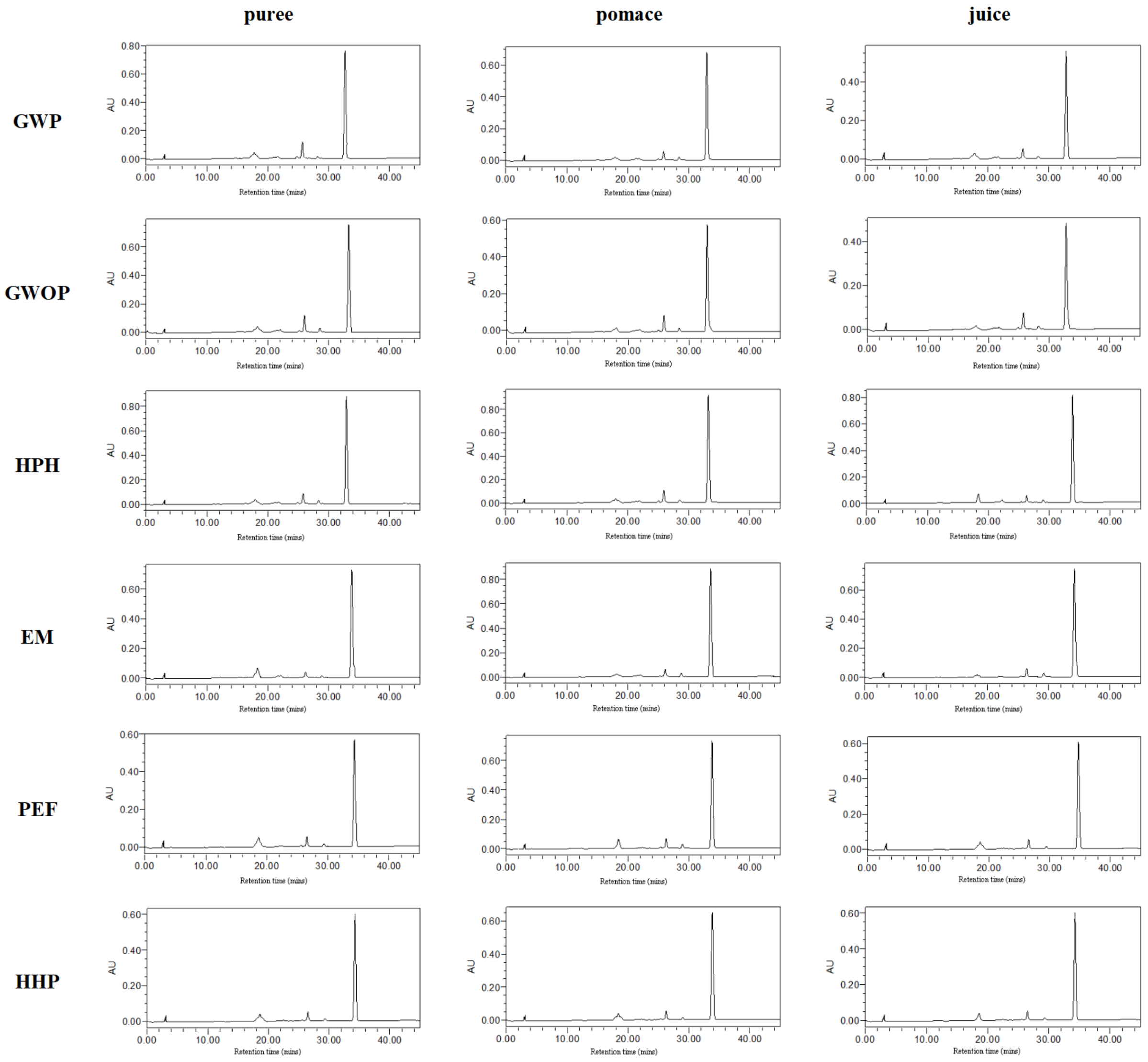

Figure S1 HPLC-DAD chromatogram of carotenoids detected in the guava samples at 450 nm.

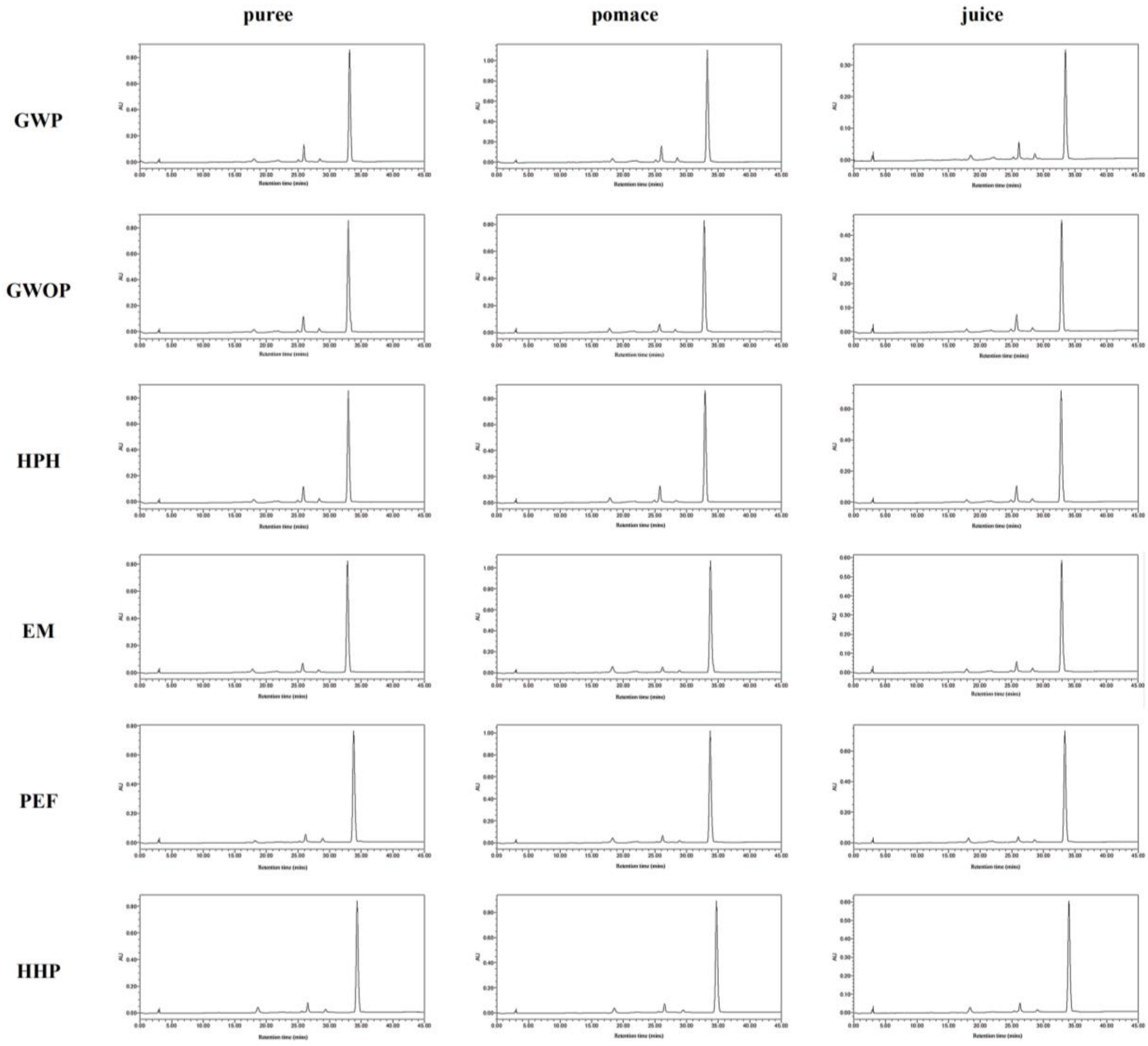

Figure S2 HPLC-DAD chromatogram of carotenoids detected in the guava samples at 470 nm.
